# Navigating the Unseen: Healthcare Support Staff’s Perception of Existential Loneliness Among Older Migrants

**DOI:** 10.1177/23333936251375459

**Published:** 2025-09-16

**Authors:** Jonas Olofsson, Ann-Cathrine Bramhagen, Margareta Rämgård, Katarina Sjögren-Forss

**Affiliations:** 1 Malmö University, Sweden

**Keywords:** existential loneliness, focus group, health care support staff, older migrants, Sweden

## Abstract

Existential loneliness is a profound sense of loneliness that arises when confronting existential questions, often intensified by the awareness of mortality. For older migrants, this experience may be deepened by feelings of exclusion and a disrupted sense of belonging. While existential loneliness has been explored in general older populations, little is known about how it is perceived and addressed by healthcare support staff working with older migrants. This study explores healthcare support staff’s perceptions of existential loneliness among older migrants and the conditions necessary to address their needs. Using focus group methodology, analysis of data generated the overarching theme of “Navigating the unseen,” capturing the complexity of recognizing and responding to existential loneliness. Three subthemes illustrate how HSSs perceive and respond to experiences of EL among older migrants. The first two—(a) “Feelings of exclusion contribute to the experience of EL” and (b) “The ongoing quest for belonging”—highlight how participants perceived older migrants’ experiences of EL. The third subtheme, “Aspects influencing HSSs’ engagement with older migrants experiencing EL,” captures factors that shape HSSs’ capacity to support this population. This study highlights the complex challenges staff face in addressing existential loneliness in this population. To meet these needs, staff require time, training, and managerial support. Building trustful relationships, fostering cultural and religious inclusivity, and integrating psychosocial care are essential to support older migrants in finding meaning and belonging in later life.

## Background

The care of older persons by healthcare support staff (HSS) involves a diverse range of personnel and individuals. This care is intended to be holistic, addressing not only the physical needs of older persons but also their mental, social, and existential needs (Abdi et al., 2019), including needs related to existential loneliness (Chung et al., 2020; Olofsson et al., 2021; Sjöberg et al., 2018). The holistic approach is based on the understanding that all these aspects of a person are interconnected, and addressing them can lead to better health outcomes, whether the individual resides at home or in a nursing home. HSS face several challenges in their care of older persons, including time constraints, inadequate training (Pessin et al., 2015; Tornoe et al., 2015), and personal discomfort (Browall et al., 2014). At the same time, older persons have a fundamental need to maintain and build interpersonal connections as they age. When care delivery becomes excessively task-oriented, neglecting the relational aspects that foster meaningful interactions, it can contribute to older persons feeling lonely and disconnected (Svanström et al., 2013).

Loneliness is a deeply subjective and contextually embedded experience, as emphasized by Dahlberg et al. (2007), who argue that it must be understood through a lifeworld perspective. From this view, loneliness is not merely an internal feeling, but a lived experience shaped by the individual’s relationship to the world, others, and themselves. It is a phenomenon that reveals itself through the person’s embodied and existential being-in-the-world, and thus resists simplistic categorization (Dahlberg et al., 2007). Social, emotional, and existential loneliness are three primary forms of loneliness (Mansfield et al., 2021), each representing a unique dimension of human disconnection. Social loneliness is characterized by a perceived disconnection from others due to a lack of social connections. Emotional loneliness is associated with profound social isolation and a lack of meaningful relationships (Mansfield et al., 2021). Existential loneliness (EL) is a multifaceted and often contested concept, with varying definitions across disciplines and contexts. While there is no universally accepted definition, EL is commonly understood as a profound sense of isolation that arises from confronting fundamental existential concerns, such as the meaning of life, the inevitability of death, and the experience of being ultimately alone in the universe. EL can also be defined as a profound sense of emptiness, sorrow, and longing that arises from the realization of human fundamental separateness (Gil Álvarez et al., 2023). This definition emphasizes the unbearable feeling of being fundamentally alone as a human being (Gil Álvarez et al., 2023). In this study, we adopt a definition of EL as a form of loneliness that emerges from an awareness of one’s mortality and a perceived disconnection from others, the world, or a greater whole (Bolmsjö et al., 2018; Ettema et al., 2010).

Existential questions are rooted in existentialism, a philosophy that explores issues concerning the meaning of life, death, freedom, and human existence. Existentialism emphasizes that individuals are free to create their own meaning and must take responsibility for their choices and actions (Baumeister, 1991). Lack of meaning leads to feelings of emptiness and despair further describing it as a state of existential vacuum (Frankl, 1985). The human being is urged to confront one’s own mortality and live authentically, despite life’s uncertainties and changes. Besides responsibility, Karl Jaspers also focused on individual freedom, and what he called ’limit situations,’ where humans are confronted with the ultimate conditions of life, such as death, guilt, and struggle (Jaspers, 1971). Existential questions accompany humans throughout life, becoming more pertinent at certain times. The realization of life’s fragility can lead to a sense of loneliness, especially when we recognize that we must navigate through a seemingly meaningless universe on our own (Baumeister, 1991). An involuntary migration can be a limit situation that can trigger the experience of EL (Olofsson et al., 2021).

Addressing EL is crucial for HSS when caring for older persons, yet it can be challenging to identify (Edberg et al., 2023; Sundström et al., 2019). Although HSS play a vital role in addressing the EL of older persons, they often struggle to meet these existential needs due to their own fear and insecurity, which can create a barrier between them and the older person (Edberg et al., 2023; Sundström et al., 2019), impacting the quality of care provided (Sundström et al., 2018; Svanström et al., 2013). The care environment also significantly influences HSS’s ability to address the existential needs of older persons (Sundström et al., 2019). For example, a familiar environment helps older persons preserve their identity (Sundström et al., 2019), and quiet spaces and nature views can help induce hope and support existential well-being (Roessler et al., 2020), thereby reducing EL and making it easier for HSS to engage with the older persons’ existential needs.

Sweden has received migrant streams from various countries and approximately 20.3% of Sweden’s population consists of individuals born abroad (OECD, 2024). The most represented countries among migrants are Syria, Iraq, and Finland. Among the older migrants, many originate from Finland, Greece, and the former Yugoslav countries. These groups migrated to Sweden during the post-war period to work (OECD, 2024). Due to multiple challenges, such as unequal opportunities to participate in social contexts (Kainradl, 2024), as well as language and cultural barriers (Olofsson et al., 2021), older migrants are considered a vulnerable group at risk of experiencing EL. Additional triggers for EL among older migrants include a lack of attachment to both place and people (Olofsson et al., 2024). EL sometimes manifests as the older migrants’ lifeworld’s collide, which can happen during the initial stages of the migration process. Furthermore, older migrants experience EL when they are unable to practice their religion (Olofsson et al., 2024), when visiting their country of origin is no longer feasible, or when contemplating death and dying in a foreign country (Olofsson et al., 2021).

Even though existential questions often remain in the background of individuals’ thoughts until triggered by significant life events (Bolmsjö et al., 2018; Karlsson et al., 2014; Sjöberg et al., 2022), HSS need the ability to recognize and respond to older migrants’ expressions of EL, regardless of cultural or contextual differences, in order to provide holistic care. However, to the best of our knowledge, there is limited research on how HSS perceive and manage EL in this population. This study therefore aimed to explore healthcare support staff’s perception of existential loneliness among older migrants and the conditions necessary to meet their needs.

## Method

This study was based on focus group methodology as described by Krueger and Casey (2015). Focus group methodology is a qualitative method that focuses on the shared experiences of the group and the collective understanding of participants’ perceptions (Ivanoff & Hultberg, 2006; Kitzinger, 1994, 1995). Furthermore, the use of focus group methodology emphasizes that knowledge is constructed in interaction with others, that is, it is a social constructivist approach (Krueger & Casey, 2015). Using the focus group method also shifts the power focus from the researcher to the participants, which affects the dynamic in the group and highlights that the participants are the experts of the subject in question (Krueger & Casey, 2015).

### Study Setting and Context

The study took place in the southern part of Sweden during 2023 to 2024. In Sweden, care for older persons is the responsibility of municipalities and run either publicly or privately. Care can be provided in the home of older persons, or in nursing homes when they are in need of around-the-clock access to skilled nursing care. In nursing homes, care is most often provided by assistant nurses. Older persons living at home have the possibility of attending activities in so-called senior citizen centers, where they can, among other things, congregate, attend physical activities, and experience fellowship. Senior citizen centers are most often managed by elderly pedagogues, social workers, or assistant nurses.

### Sample and Recruitment

Five focus groups were conducted with a total number of 21 HSS (Table 1). Nineteen women and two men participated, their age varying between 25 and 65 years. There was a variation in the length of work experience from 3 to 40 years. Information about participants’ country of birth was not systematically collected. However, based on self-disclosures during the interviews, twelve participants were identified as having a non-Swedish background originating from Europe, Middle East, Africa and South America. It is not known whether these individuals were born abroad or are second-generation migrants. To create dynamic focus groups, both homogeneity and heterogeneity were considered. Homogeneity concerns sharing similar experiences, which in this study means that participants had shared experiences in caring for older migrants. Heterogeneity is about variation in the collected data (Krueger & Casey, 2015). Therefore, there was a striving for variation in age, gender, type of healthcare professional, and length of time working in care for older persons. Elderly pedagogues, and assistant nurses with special training in care for older persons, psychiatric, or dementia care, either working in nursing homes, home care, or senior citizen centers, were invited to take part in the study.

**Table 1.** Focus Group Characteristics.

Focus group	Context	Number of participants	Number of participating women/men	Age, mean (range)	Year in profession, mean (range)	Length of group discussion (minutes)
1	Public	4	3/1	51 (33–58)	31 (9–40)	84
2	Private	4	3/1	41 (25–57)	6 (3–15)	45
3	Private	4	4/0	54 (46–65)	20 (6–25)	42
4	Private	4	4/0	55 (43–65)	27 (14–38)	69
5	Public	5	5/0	47 (29–56)	18 (9–33)	60

### Recruitment Process

In the first step of the recruitment process, after having received permission from the relevant branch heads, the coordinator of care in each nursing home or senior citizen centers was contacted and an email with information about the study was sent out. The invitation and information letter contained details about the study and a description of EL. The coordinator was asked to forward the invitation for participation in the study. The coordinator then contacted the first author with the names and email addresses of prospective participants. In this step, thirteen HSS agreed to participate. The recruitment process was expanded by sending out emails to coordinators in nursing homes from different municipalities in the southern part of Sweden. Additionally, two more nursing homes in two different municipalities agreed to participate, and the recruitment process led to another nine participants being recruited. In total, five focus groups were conducted.

### Focus Groups

Data were collected through the focus group method, and an interview guide, with open questions focusing on the HSS perception of older migrants’ EL, was used. Prior to the main study, a pilot study was conducted to evaluate the research questions (Malmqvist et al., 2019). No changes to the questions were made, but the pilot study was not included in the main study. Three of the focus groups were held outside the HSS workplaces and two of the focus groups at the HSS workplaces. The group were guided by a moderator. The role of the moderator was to create an open and friendly atmosphere so that the participants could feel free to express their opinions. It was important for the moderator not to interfere in the focus group discussion but simply to moderate. An observer (KSF) was present in two of the focus groups, taking notes, provide feedback to the moderator regarding group dynamics and facilitation as well as adding clarifying questions. Due to unforeseen events, the observer was unable to attend the remaining sessions, and it was not possible to arrange a replacement on short notice. The participants were placed in a circle around a table, and the moderator started the discussion by explaining the aim of the study and explaining briefly what EL is about. The moderator then followed the proposed interview guide, starting with an open question about the HSS experience of older persons and EL. Before the focus group sessions began, the information about the study and the description of EL was reiterated, and participants were given the opportunity to ask questions. The moderator emphasized that the aim was to understand their perceptions of EL, clarifying that there were no right or wrong answers. It was also stressed that differing opinions were expected and welcomed in the discussion. EL was reiterated as a deeper sense of loneliness that one can experience despite having family and friends nearby. It is a feeling that can come and go. The opening question was: “What is the reason for you working in care for older persons?” When thinking about the older migrants you care for, can you recall encountering older migrants who have experienced EL? How did it manifest? Can you provide an example? Further questions were about their experience of older migrants and their EL. For example, the HSS were asked to think about a situation where they encounter older migrants experiencing EL, and then they were asked to answer the following question: “In your opinion, do you think there is a difference in caring for older migrants experiencing EL compared with older non-migrants? If so, how does it differ?’ The HSS talked about their experiences by describing situations from work or clinical practices. Most of the time, the discussions proceeded without the need for intervention from the first author. It was a respectful discussion where the HSS listened to each other and there was not any participant being more dominant in the discussions. However, on occasions where clarification was needed, the moderator would pose a question to gauge whether others agreed or disagreed with a particular statement. The group discussions were audiotaped and transcribed verbatim and lasted between 42 and 84 min (Table 1).

### Data Analysis

The analysis of the focus group discussions was conducted as described by Krueger and Casey (2015). Fieldnotes and observations were made during the focus groups and were used when analyzing the data. After transcription, the interviews were listened to, and the transcripts were read thoroughly several times by the first author (JO) to get an overall sense of the raw data. The aim of the study and the participants collective understanding of the topic were in focus when analyzing the data. Text units answering the aim of the study were identified, condensed, and coded and sorted into categories. The first author (JO) then went through the material again to make sure the aim of the study was in focus and that important data were not missing. To get a contextual meaning of the data, all authors read through the material several times and all authors were involved in the analysis process. The main theme and subthemes were constructed through this iterative process, involving both inductive reasoning and analytical prioritization, as recommended by Krueger and Casey (2015). Themes were prioritized based on their relevance to the research question, recurrence across groups, and the richness of the supporting data.

### Ethical Considerations

The current study strictly adhered to the Helsinki Declaration – Ethical Principles for Medical Research (World Medical Association, 2025). Permission to conduct the study was granted by the Swedish Ethical Review Authority (Reg. No. 2021-03577). Information about the study was given either verbally or in writing to relevant branch heads. Once the permission to conduct the study was established, written information was sent out by the first author (JO) to potential participants. Prior to the focus group discussion, participants were given written and oral information about the purpose of the study and assurance that their participation was voluntary and that they had the possibility to withdraw from the focus group discussion without any explanation. Before the written consent was collected, there was a possibility for the participants to ask questions. The written information about the study contained a phone number to a contact person outside the study in case any participants felt any discomfort related to the focus group discussion and needed someone to talk to afterwards. Participants were informed about the limits of confidentiality inherent in focus group settings and were asked to respect each other’s privacy and not disclose any information shared during the discussions. The confidentiality of the participants was maintained through protection of the data collected.

## Result

The analysis generated one overarching theme and three sub-themes. The overarching theme, *Navigating the unseen*, encapsulates the complex and often hidden experiences of EL among older migrants. This theme reflects the challenges faced by HSS in recognizing and addressing EL, which may not be immediately visible or easily understood. Three subthemes illustrate how HSSs perceive and respond to experiences of EL among older migrants. The first two—(a) Feelings of exclusion contribute to the experience of EL and (b) The ongoing quest for belonging—highlight how participants perceived older migrants’ experiences of EL. The third subtheme, Aspects influencing HSSs’ engagement with older migrants experiencing EL, captures factors that shape HSSs’ capacity to support this population.

### Feelings of Exclusion Among Older Migrants Lead to the Experience of EL

Feelings of exclusion emerged as a central aspect of existential loneliness (EL) among older migrants. The HSS described how older individuals often experienced a sense of not belonging, which was reinforced by language barriers, cultural differences, and unfamiliarity with the healthcare system in the host country. These factors contributed to a form of involuntary isolation that intensified the experience of EL.

One HSS explained:

Yes, and also a sense of exclusion. It happens when someone sits there and feels, “Oh, I don’t understand the language.” That can make a person feel very lonely, just sitting there. . . (P1, Focus group 2)

The HSS noted that older migrants often struggled to adjust to the structure and expectations of the healthcare system, which differed from those in their countries of origin. This unfamiliarity created a sense of alienation and marginalization, particularly when communication was hindered by language loss or cognitive decline.

And then, as they grow older, they begin to lose their Swedish, perhaps due to dementia, and Finnish is what remains. But then no one understands them. And there’s this anxiety, even before it happens, about what will come. (P1, Focus group 1)

In some cases, this led to misunderstandings and distressing experiences, such as believing that care staff were speaking a foreign language or that they were surrounded by strangers. The presence of someone who spoke their native language could bring immediate relief and a sense of calm, highlighting the crucial role of language in mitigating EL.

Then someone came in who spoke Finnish, and suddenly there was a sense of calm. Language plays a crucial role, and not having it in one’s daily life. . . (P1, Focus group 1)

In addition to these feelings of exclusion, the HSS observed that older migrants often expressed their existential loneliness in vague or indirect ways. Their expressions were described as elusive, as if they were carrying a secret, which made it difficult for the HSS to identify specific signs or fully understand their emotional state.

She was extremely anxious—constantly asking, “Who is working today? There were so many questions all the time. (P3, Focus group 2)

This vagueness, combined with cultural norms around emotional expression, contributed to a sense of invisibility and made it challenging for healthcare staff to respond adequately to the older migrants’ existential needs.

### The Quest for Belonging

The HSS perceived that there was something missing in the older migrants’ life, but both the older migrants and the HSS struggled to define the elusive “something” contributing to dissatisfaction. According to the HSS, this dissatisfaction originated in experiences of life being different from life in the country of origin, making the older migrants feel rootless and that they did not belong, which triggered the experience of EL that led to the older migrants longing for home. The HSS concurred that this longing was indicative of a desire for the security and familiarity of their past lives in their home country, rather than a desire to return to their country of origin.

*P1:* When living in a nursing home, they are at home but not really in their own home. We say they’re at home because when they ask, “Where am I?” — “You’re at home, you live here.” But it’s not the same. They don’t think the same way; they think about their old life. (Focus Group 2)

It was also observed by the HSS that questioning life choices led to existential guilt and experiences of EL. Older migrants often wondered whether they made the right decision to migrate and speculated about what their lives would have been like in their country of origin during their later years. This questioning was partly driven by the expectations older migrants have of their next of kin. The HSS agreed that older migrants had higher expectations of care from their next of kin compared to non-migrants. As the HSS at times struggled to understand what the older migrants tried to express, they felt that their next of kin were better able to understand. The HSS stated that the older migrants often had a close, if more complex, relationship with their next of kin and that they therefore did not experience as much loneliness. However, even though the HSS experienced older migrants as not being socially lonely due to a close relationship with family and, when living in a nursing home, to fellow caretakers, the older migrants felt EL. Additionally, the absence of natural social settings, such as workplaces or the proximity of family and relatives, contributed to feelings of not belonging and being different, which triggered the experience of EL. These reflections were echoed by staff members, who discussed the limited visibility of older migrants in society and pointed to the lack of employment opportunities and natural meeting places as contributing factors.

*P1:* Something I’ve been reflecting on is that this city is such a cultural city. There are so many people from other countries, but you don’t really see them that much. // There should. . . I mean, you do meet them, but there should be more, considering there are 173 different nationalities [in the city].

*P2:* Yes, but I think when you get older and you’re no longer part of working life, I think you kind of withdraw into yourself and feel more different than you do when you belong somewhere through work or something like that. So I think that’s why — we don’t see them, but you meet them. . . if they need help, then you go on a home visit or they have home care. They feel even more different. (Focus group 1)

According to the HSS, older migrants perceive religion as more than just a belief. For many, it is a way of life that provides a sense of belonging with both people and God. The HSS stated that, regardless of whether religion or existential questions had been prominent earlier in the older migrants’ lives, these thoughts could become important to share towards the end of life. However, the HSS believed that older migrants perceive a resistance to religion in Sweden’s secular society, which could prevent them from practicing their religion, thus contributing to an increasing experience of EL.

*P2:* Sweden is so secularized. . . if you meet someone who goes to church every Sunday, the reaction is often, “Oh, this person must be a deeply committed Christian.” It’s not considered typical. But someone who goes to the mosque every Friday, that might be seen as something more than just religious practice. It’s shared. . . it’s something entirely different.

*P1:* It’s a way of life.

*P2:* Yes, the church has lost that function in Sweden. People don’t go to church on Sundays anymore, very few do. That sense of community has been lost. Whereas Islam, I don’t know, it can represent much more. It can be a way of life, a place where people meet. We work together. (Focus group 1)

Although there was a common agreement among the HSS about the importance of religion in many older migrants’ lives, there were differences in how it was approached and viewed. Some HSS said that there was no hindrance for HSS to approach older migrants’ spiritual needs. Others, however, shared experiences of resistance among HSS.

### Aspects Influencing HSS’s Engagement with Older Migrants Experiencing EL

The HSS knew older migrants had needs in relation to their EL. They reported that encountering older migrants experiencing EL added significant meaning to their work. However, they also faced substantial challenges. One major challenge was the inability to utilize their knowledge and skills effectively. The HSS working in nursing homes expressed frustration at being limited to providing fundamental physical care, such as nutrition, hygiene, and mobilization, while the emotional, social, and existential needs of older migrants were often deprioritized.

*P1:* The stress can also come from the fact that we have a lot of demands placed on us. . .

*P2:* Yes, we really do.

*P1:* . . . from above.

*All participants:* Mm.

*P1:* Partly from the managers, but also from the politicians who sit and decide how things should be. . .. . . and what it should look like, and they have no idea what it’s actually like. And then it’s like, “For this amount of money, you’re supposed to do all of this. . .” . . . and you just wonder how. And it’s like a commitment; we’re supposed to do it. That’s just how it is. And then you can’t give a hundred percent, maybe it ends up being seventy-five percent, and that doesn’t always feel good. Because you want to give more. (Focus group 4)

The HSS emphasized that being proud of one’s education and dedicated to the profession was crucial for interactions with older migrants. Most HSS further highlighted the need for deeper conversations with older migrants about existential questions as well as EL but agreed upon both having insufficient awareness of the backgrounds of older migrants and limited time, which complicated interactions and understanding of older migrants EL. Additionally, the HSS felt frustrated by the lack of trust in their professional judgment on the part of the leadership. Frequent changes in work duties and time constraints often prevented them from spending adequate time with their patients, including older migrants. They felt that they needed to spend sufficient time with older migrant persons in order to address their feelings of EL. However, the HSS working in senior citizen centers, as opposed to nursing homes, felt they had more opportunities to provide social, emotional, and existential care even if it was challenging. They also viewed their role in relation to older migrants differently, recognizing the need to build relationships and become a friend.

*P4:* I find [existential] conversations difficult. I mean, I struggle with those conversations because I don’t know how to start. Some people can do it naturally, but I find it hard. Some say they are angry because they have a loss or grief they haven’t talked about. When you open up that conversation, a lot of the anger disappears. It’s very much about building relationships. You become a friend. And then it gets easier. But it takes time. (Focus group 1)

## Discussion

A central finding in this study is the difficulty HSS experience in identifying and addressing EL among older migrants. Thus, this study reports that EL often is difficult to articulate and recognize, both for those experiencing it and for those providing care. Furthermore, the concept of EL remains theoretically complex and lacks a universally accepted definition. However, through literature, EL is consistently described as distinct from social or emotional loneliness. While social loneliness refers to the absence of social contacts and emotional loneliness to the lack of close, meaningful relationships, EL is rooted in a deeper existential awareness of one’s fundamental separateness and mortality. Ettema et al. (2010) conceptualize EL as an intolerable emptiness, sadness, and longing that arises from the awareness of one’s existential isolation, and they distinguish between EL as a condition, an experience, and a process of inner growth. Moustakas (1961) similarly describes EL as a universal and inescapable part of the human condition (Moustakas, 1961). Bolmsjö et al. (2018) emphasize the emotional and relational dimensions of EL, highlighting how it involves disconnection not only from others but also from meaning and belonging. The ambiguity surrounding the concept may contribute to its invisibility in everyday care practices, particularly when time and resources are limited. A clearer understanding of EL as a unique and deeply personal form of distress is therefore essential for developing care approaches that are sensitive to the existential dimensions of older migrants’ experiences. This challenge is partly rooted in the older individuals’ limited ability or willingness to express existential concerns. EL differs from other forms of loneliness in that it touches on the core of human existence, our awareness of mortality, loneliness, and the search for meaning (Jaspers, 1971; Yalom, 2020). EL is further not something that can be cured, but rather something that must be acknowledged and accompanied. This aligns with previous research emphasizing that EL requires a different kind of presence, one that is relational, compassionate, and attuned to vulnerability (Olofsson et al., 2021). For HSS, this presents a unique challenge, especially when working with older migrants whose experiences are shaped by cultural, linguistic, and spiritual dimensions. The findings suggest that EL is often invisible and unspoken, requiring staff to develop a sensitivity to what is not said.

One of the most powerful insights from the focus groups was the importance of relational presence. Older migrants’ feelings of meaninglessness and losses may evoke EL influencing older migrants’ thoughts about the future regardless of age or life expectancy. The HSS encounter older migrants within this context and the care provided by the HSS is to some extent based on the story of the older migrant. To establish meaningful relationship where there is an ongoing learning about a person’s cultural background, it is crucial for the HSS to be other-oriented (Stubbe, 2020). The HSS emphasized the value of building trust and becoming a friend, rather than merely performing tasks. This relational approach is essential in addressing EL, as many older persons do not initiate existential conversations themselves. Instead, it often requires the initiative of a compassionate other, someone who dares to be present in the face of vulnerability. The metaphor of the HSS as a friend underscores the emotional labor involved in this work. Yet, this ideal is often challenged by structural barriers such as time constraints, high workloads, and limited cultural knowledge.

Another key theme was the experience of exclusion and the longing for belonging among older migrants. Some HSS perceived no major difference in EL between older migrants and other older adults, while others highlighted specific cultural and contextual factors such as language barriers, religious practices, and family dynamics that shaped how EL was experienced and expressed. These findings align with previous research suggesting that while EL may be a universal phenomenon, its manifestations are culturally and socially mediated (Chung et al., 2020; Olofsson et al., 2021, 2024). Older migrants’ states that regardless of how long they had lived in the host country, many expressed feelings of being different or not fully accepted. Further, the experience of exclusion may give rise to a longing for belonging and a search for meaning (Chung et al., 2020; Olofsson et al., 2021, 2024). As Frankl (1985, 2020) emphasizes, the search for meaning in life can be deeply personal and shaped by one’s attitude toward suffering.

The findings also highlight the central role of religion in the lives of many older migrants, as perceived by HSS. Religion was described as a significant source of comfort and a way of coping with EL. This aligns with previous research showing that religious beliefs can offer meaning, identity, and emotional support in times of existential distress (Olofsson et al., 2024). For many older migrants, religion is not only a private belief system but a public practice that shapes their worldview and daily life (Guveli & Platt, 2023; Spännäri & Laceulle, 2021). This may contrast with Western norms, where religion is often seen as a private matter. However, HSS expressed uncertainty about how to address the spiritual needs of older migrants, particularly when they lacked knowledge of diverse religious traditions. This reflects a broader challenge in multicultural care settings, where religious literacy, the ability to understand and respectfully engage with different religious practices, is increasingly important (Lavik et al., 2021). At the same time, staff may worry about overstepping professional boundaries, which can create hesitation in addressing spiritual concerns (Georgeou et al., 2023).

Finally, the findings underscore how EL often emerges in what Jaspers (1971) calls limit situations, unavoidable life events such as death, suffering, or guilt that confront individuals with the boundaries of existence. These situations tend to become more prominent in later life, particularly when individuals face illness, loss, or the approach of death (Saarelainen et al., 2023). When such questions remain unanswered or unacknowledged, they may give rise to a deeper form of loneliness. For older migrants, these limit situations may be intensified by the experience of migration, which can involve loss of homeland, identity, and belonging (Olofsson et al., 2021). Thus, given the complexity of addressing existential loneliness among older migrants, HSS plays a crucial role in this process. This study illustrates how HSS are both witnesses to and engaged in these existential struggles faced by older migrants.

## Methodological Considerations

The idea behind focus groups is to generate discussion and thereby get a collective understanding of HSS views, that is, a collective construction of meaning (Ivanoff & Hultberg, 2006; Krueger & Casey, 2015). A crucial feature for the moderator is to stimulate interaction between participants in order to create a discussion (Ivanoff & Hultberg, 2006; Krueger & Casey, 2015). Some researchers argue that there should be 6 to 12 participants in each group (Krueger & Casey, 2015), while others argue for less (Guest et al., 2017). Thus, an issue to discuss with regard to this study may be the limited number of participants in each group. We found, however, that the discussions were dynamic, and the outcome of the discussions depended more on the involvement of the participants in each group than on the actual number of participants. We also found that the focus group discussions allowed the participants to verbalize and share their experiences in an area they considered important. To obtain a broad representation of the target group and at the same time create an atmosphere that generates discussion, it is necessary to consider the heterogeneity and homogeneity within the groups. What united our participants, homogeneity, was their common experience of being HSS encountering older migrants in their daily work, and the heterogeneity was based on the fact that they were from different contexts within care for older persons, thus representing different disciplines. The heterogeneity was, furthermore, based on differences in age, country of origin, gender, and years in the profession (Krueger & Casey, 2015).

The moderator played a crucial role in leading the discussion, encouraging all HSS in the focus group, and managing group dynamics, as each focus group was unique. It is, further, important to note that an observer was present during two of the focus group sessions, while the remaining three sessions were conducted without an observer. The presence of an observer can influence the dynamics and interactions within the group, potentially affecting the data collected. However, it is our understanding that the variation in observer presence across sessions did not affect the findings. It was also important to create an atmosphere where HSS felt safe sharing their opinions, which was achieved in all focus groups.

## Implication

To move forward, care systems must recognize existential loneliness not as an abstract or peripheral concern, but as a core dimension of human well-being, one that intersects with identity, belonging, and the right to be seen. Given the results of the study, there is a need for education to better understand and address the EL of older migrants. The importance of building relationships and offering empathetic support is central. HSS staff need to develop skills in creating trustful relationships with older migrants to address their EL, which also includes support from management. Management must prioritize and allocate resources to enable this work. For HSS to provide adequate support, sufficient resources, including time and personnel, are required. The older migrants need psychosocial support as they may experience a combination of social isolation and existential loneliness. Therefore, HSS needs to integrate psychosocial support into their work to help these individuals find meaning and context in life. Finally, care environments need to be adapted to be culturally and religiously inclusive. This may involve creating spaces for religious practices and ensuring that staff are aware of and respect these needs.

## Conclusion

The study highlights the significant challenges faced by HSS in addressing older migrants experience of EL. It underscores the necessity for comprehensive education and training to enhance the understanding and management of older migrants’ existential needs. The challenges HSS face are related to time restraint, lack of knowledge in encountering older migrants’ existential issues including EL and feelings of not being trusted with the work. HSS staff must develop relational skills and receive support from management, which should prioritize and allocate adequate resources, including time and personnel, to facilitate this work. The integration of psychosocial support is essential, as older migrants often experience a combination of social isolation and existential loneliness.
